# Variations in sexual function after laparoendoscopic single-site hysterectomy in women with benign gynecologic diseases

**DOI:** 10.1515/med-2023-0761

**Published:** 2023-07-31

**Authors:** Jingyun Xu, Qiuping Qian, Mulan Ren, Yang Shen

**Affiliations:** Department of Obstetrics and Gynecology, Zhongda Hospital, School of Medicine, Southeast University, Nanjing 210009, Jiangsu, China; Department of Gynecology and Obstetrics, Wuxi Hospital of Maternal and Child Health Care, Wuxi 214000, Jiangsu, China

**Keywords:** laparoendoscopic single-site hysterectomy, sexual function, female sexual function index, benign diseases

## Abstract

Laparoendoscopic single-site surgery (LESS) has become a novel minimally invasive approach applied as an option to perform hysterectomy. The aim of the study was to evaluate the influence of LESS hysterectomy on the sexual function in women with benign gynecologic indications. From October 2016 to May 2021, a total of 486 premenopausal, sexually active women were eligible. Female sexual function index (FSFI) was used to assess sexual function preoperatively and 6, 12 months postoperatively. Total FSFI score ≤26.55 indicated female sexual dysfunction (FSD). Compared with pre-operation, each subdomain and total FSFI scores increased at 6 (all *p* < 0.05) and 12 months (all *p* < 0.001). Prevalence of FSD decreased at 6 (30 vs 39.9%, *p* = 0.002) and 12 months (27 vs 39.9%, *p* < 0.001). In patients with preoperative FSD, each subdomain and total FSFI scores improved at 6 and 12 months (all *p* < 0.001), while decreased at 6 months (*p* < 0.001) and had no significant difference at 12 months (*p* = 0.54) in patients without preoperative FSD. These results suggest that LESS hysterectomy has a significant positive effect on the sexual function in women with benign gynecologic diseases, especially those with preoperative FSD.

## 1 Introduction

Hysterectomy is one of the most frequent surgeries in gynecology worldwide [1], among which 70% are performed on the women with benign indications [2]. Hysterectomy can be performed using vaginal, laparoscopic, and abdominal approaches or a combination of these techniques [3]. In the last few decades, more and more less-invasive endoscopic techniques have been used in hysterectomy with technical developments.

Laparoendoscopic single-site surgery (LESS), a novel minimally invasive surgery, appears to be feasible and safe to perform in a variety of gynecologic diseases [4]. A previous study done in Korea showed that 80% of the hysterectomies were performed via LESS [5]. Additionally, a recent meta-analysis performed by Michener et al. demonstrated that LESS hysterectomy was feasible, safe, and equally effective compared with the conventional multiport laparoscopic hysterectomy [6]. However, performing a hysterectomy can cause different injuries to several anatomical structures, innervation, and blood supply of the pelvic floor, resulting in various complications [7].

It is known that sexual function is a major cause of the women’s concern for scheduled hysterectomy and may be a cause of anxiety prior to surgery [8]. Although the effect of hysterectomy on female sexual function (FSF) has been investigated for a long time, it is still undefined on whether FSF improves or worsens following hysterectomy. There is a study suggesting that hysterectomy does not have any effect on the FSF [9]. Thakar found that a significant minority of women suffered from sexual dysfunction after hysterectomy [10], while positive influences on postoperative sexual function were also reported in other studies [11,12].

Until now, most studies reporting the effects of hysterectomy on FSF focus on use of vaginal, laparoscopic, and abdominal approaches [13]. There are few studies exploring the impacts of LESS hysterectomy on FSF. The FSF following LESS hysterectomy may be different because an adequate margin of the vagina is needed to suture the vaginal stump, avoiding the collision of multiple instruments through a single small incision. Therefore, the purpose of this study was to investigate the effect of LESS hysterectomy on the sexual function in sexually active premenopausal women with benign disorders.

## 2 Materials and methods

### 2.1 Participants

This was a prospective, descriptive, observational study. Patients undergoing LESS hysterectomy at the Zhongda Hospital, Southeast University, China between October 2016 and May 2021 were included.

The study included premenopausal, sexually active women (i.e., at least one episode of intercourse in the 3 months before surgery), who were aged between 18 and 50 years, and who underwent LESS hysterectomy due to benign gynecological indications (uterine myoma, adenomyosis, dysfunctional uterine bleeding, etc.). The exclusion criteria were as follows: (1) homosexuality (the female sexual partner), (2) changes in sexual partners during the study period, (3) conversion to laparotomy or laparoscopy due to intraoperative complications, (4) presence of a previous or concomitant surgery (pelvic organ prolapsus or anti-incontinence surgery), (5) postoperative malignant pathology, (6) history of gynecologic malignancy, (7) current or past psychiatric diseases and intellectual impairment, and (8) failure in attending the follow-up visit.


**Ethical approval:** The study protocol was reviewed and approved by the Zhongda hospital s Institutional Review Board (protocol registration no. 54/PB/2017). All participants provided their informed consent.

### 2.2 Procedures for LESS hysterectomy

LESS hysterectomy was performed through a multichannel single trocar inserted in the umbilicus using an open technique, with 2 cm cutaneous incision. Surgical steps followed identical steps reported by Fanfani et al. [13].

### 2.3 Data collection and female sexual function index (FSFI) assessment

Demographic and clinical data were collected from the medical record and recorded in a prospective database, including age, body mass index (BMI), parity, number of vaginal deliveries, educational levels, history of previous abdominal surgery, and indications for surgery.

The FSFI of participants who agreed to participate was assessed preoperatively (1–2 weeks before surgery, T1), 6 and 12 months after surgery (T2 and T3), respectively. The FSFI is an externally validated self-report questionnaire used to assess FSF [14]. The FSFI consists of 19-item questions that measure six domains of sexual functioning, which are scored and summed to arrive at a total score that may range from 2.0 to 36.0. The minimum and maximum scores for each domain are as follows: desire (range 1.2–6), arousal (range 0–6), lubrication (range 0–6), orgasm (range 0–6), satisfaction (range 0.8–6), and pain (range 0–6). Higher scores in the FSFI indicate better functioning. A cut-off score of 26.55 has been validated to discriminate between sexually functional and dysfunctional women, with those scoring 26.55 or below being considered likely to have female sexual dysfunction (FSD) [15].

In this study, data from questionnaires were collected at scheduled clinic visits, alternatively, through the phone if patients failed to complete questionnaires during scheduled appointments. Meanwhile, as a complete FSFI was necessary to calculate the overall score, the participants who did not answer all the questions were excluded.

### 2.4 Statistical analysis

Data were analyzed using SPSS 23.0 (SPSS Inc., Chicago, IL, USA). The Shapiro–Wilk test was used to determine normalization. Continuous variables were presented as mean ± standard deviations, while categorical data were presented as counts and percentages. A repeated measures analysis of variance was applied to compare the differences between each postoperative and preoperative FSFI. In case of violation of sphericity, the Greenhouse–Geisser correction was assumed. The *post-hoc* Bonferroni test was used for pairwise comparison. Fisher’s exact test or *χ* ^2^ test was used to determine the differences in the prevalence of FSD at different time points. *p*-Values < 0.05 were considered statistically significant.

## 3 Results

### 3.1 Participant demographics

Between October 2016 and May 2021, 568 women were scheduled for LESS hysterectomy due to benign gynecological indications, among whom 486 cases were finally included into the analysis due to 18 cases without written informed consent and 64 cases with missing follow-up data at least at one time point. The demographic and clinical characteristics of the 486 participants are listed in Table 1. The median age was 44.74 ± 3.99 years. The median BMI was 24.01 ± 3.49 kg/m^2^, and the most common indication for surgery was myoma uteri (34%).

**Table 1:** Demographic and clinical characteristics of 486 participants undergoing LESS hysterectomy

Characteristics	Value (*n* = 486)
Age^a^ (years)	44.74 ± 3.99
BMI^a^ (kg/m^2^)	24.01 ± 3.49
Parity^b^	
0	9 (1.8%)
1–3	463 (95.3%)
>3	14 (2.9%)
Number of vaginal deliveries^b^	312 (64.2%)
Educational levels^b^	
Basic	43 (8.9%)
Secondary	87 (17.9%)
Student	211 (43.4%)
Higher	145 (29.8%)
Previous history of abdominal surgery^b^	263 (54.1%)
Indications for surgery^b^	
Myoma uteri	165 (34.0%)
Adenomyosis	98 (20.2%)
Dysfunctional uterine bleeding	78 (16.0%)
Endometrial hyperplasia	45 (9.2%)
Cervical intraepithelial neoplasia grade III	86 (17.7%)
Others	14 (2.9%)

^a^Data are expressed as mean ± standard deviations. ^b^Data are expressed as the count and percentage. Abbreviations: BMI, body mass index (kg/m^2^); LESS, laparoendoscopic single-site surgery.

### 3.2 Changes of sexual function

**Figure 1:**
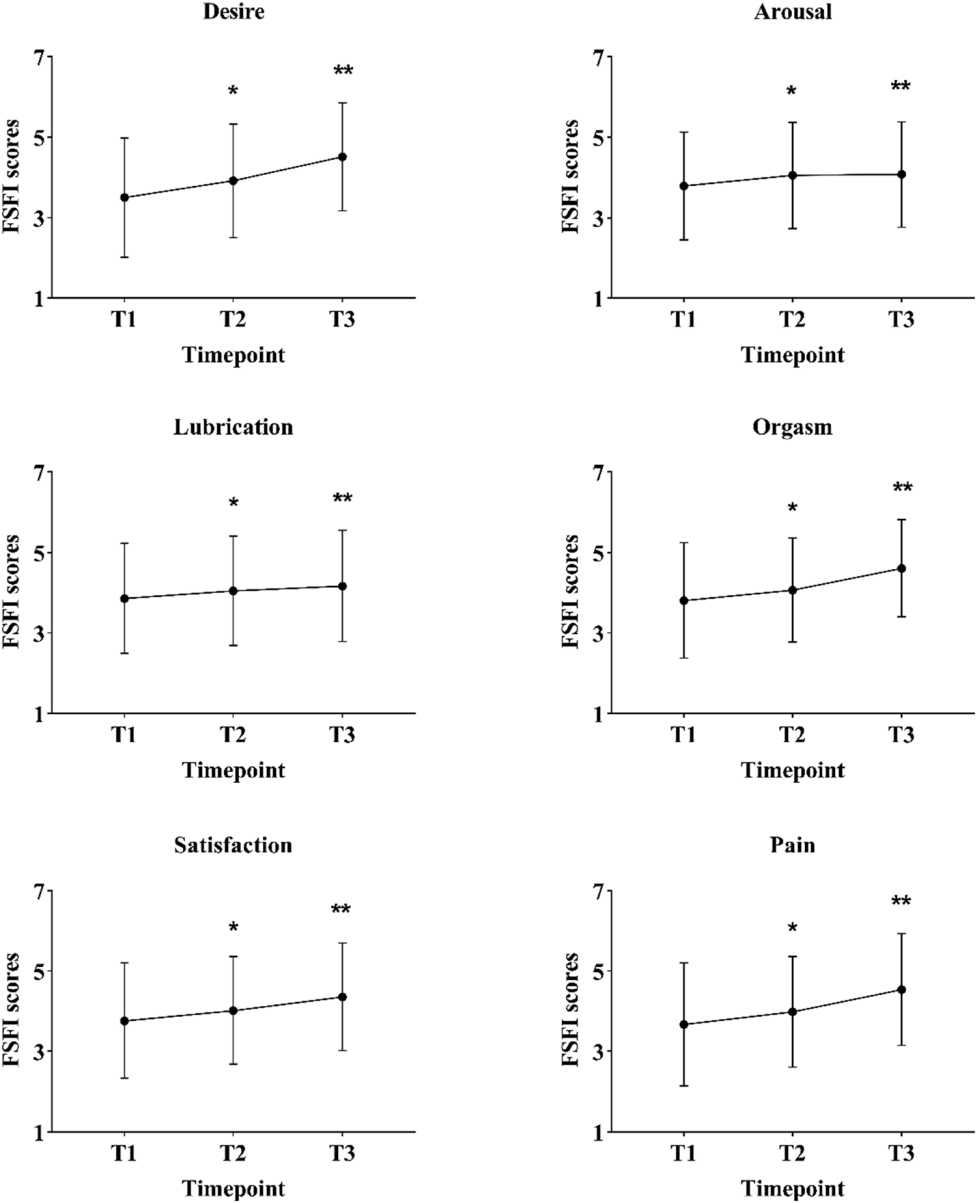
Changes of each FSFI subdomain in all patients undergoing LESS hysterectomy at different time points. Abbreviations: FSFI, female sexual functional index; T1, pre-operation; T2, 6 months post-operation; T3, 12 months post-operation. *p < 0.05 represents the difference between T1 and T2. **p < 0.05 represents the difference between T1 and T3.

Changes of sexual function for all participants are shown in Figure 1. A statistically significant increase in desire (*F* = 66.537; *p* < 0.001), arousal (*F* = 7.957; *p* < 0.001), lubrication (*F* = 10.640; *p* < 0.001), orgasm (*F* = 50.752; *p* < 0.001), satisfaction (*F* = 24.362; *p* < 0.001), and pain (*F* = 47.296; *p* < 0.001) was observed along with time variation, including between T1 and T2 (*p* < 0.001, *p* = 0.003, *p* = 0.006, *p* = 0.032, *p* = 0.009, and *p* = 0.002, respectively) and T1 and T3 (all *p* < 0.001).

**Figure 2:**
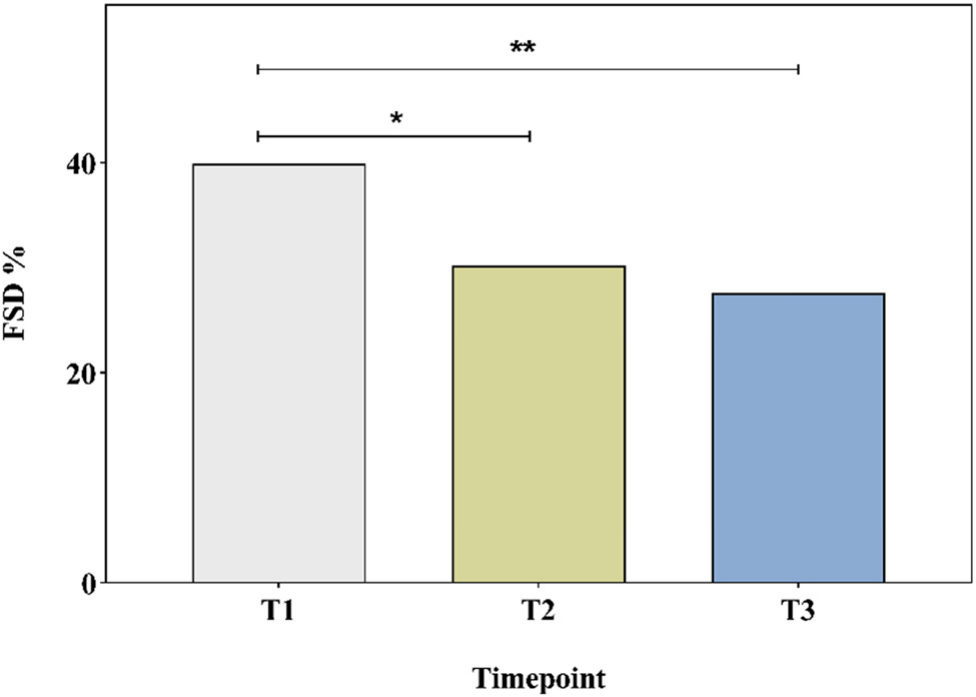
Prevalence of FSD in all patients undergoing LESS hysterectomy at different time points. Abbreviations: FSD, female sexual dysfunction; T1, pre-operation; T2, 6 months post-operation; T3, 12 months post-operation. *p < 0.05 represents the difference between T1 and T2. **p < 0.05 represents the difference between T1 and T3.

FSD was diagnosed in 194 (39.9%) of 486 patients preoperatively, and declined significantly to 30.2% at T2 (*p* = 0.002) and 27.6% at T3 (*p* < 0.001). The prevalence of FSD is presented in Figure 2.

**Figure 3:**
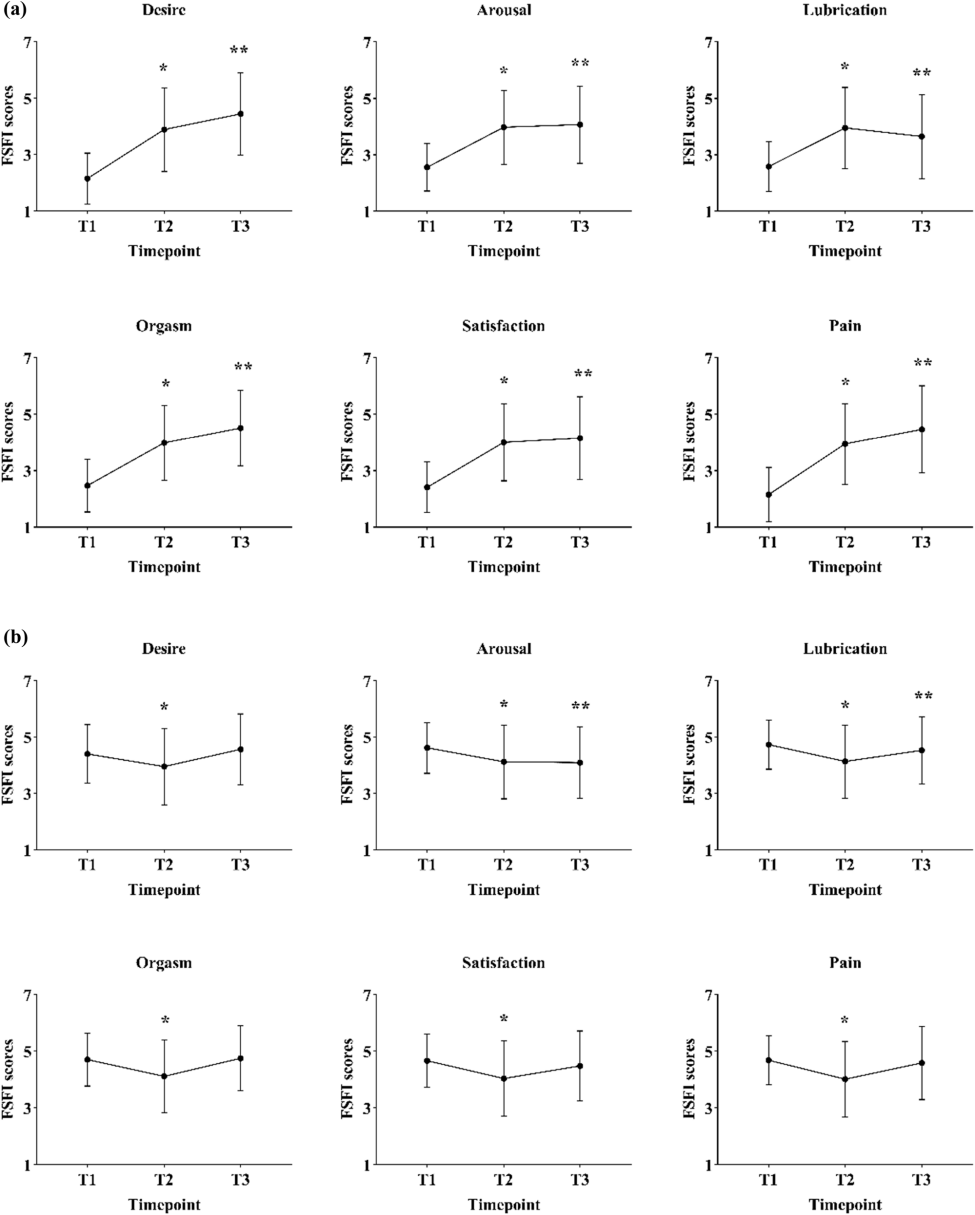
Changes of each FSFI subdomain in patients with preoperative FSD (a) and those without preoperative FSD (b) at different time points. Abbreviations: FSD, female sexual dysfunction; T1, pre-operation; T2, 6 months post-operation; T3, 12 months post-operation. *p < 0.05 represents the difference between T1 and T2. **p < 0.05 represents the difference between T1 and T3.

In patients with pre-FSD, the FSFI in all subdomains, including desire (*F* = 211.501; *p* < 0.001), arousal (*F* = 138.464; *p* < 0.001), lubrication (*F* = 97.348; *p* < 0.001), orgasm (*F* = 154.554; *p* < 0.001), satisfaction (*F* = 113.629; *p* < 0.001), and pain (*F* = 160.129; *p* < 0.001), increased significantly from T1 to T3 (T1 vs T2 all *p* < 0.001; T1 vs T3 all *p* < 0.001) (Figure 3).

In patients without pre-FSD, the FSFI in all subdomains, including desire (*F* = 20.546; *p* < 0.001), arousal (*F* = 20.649; *p* < 0.001), lubrication (*F* = 25.188; *p* < 0.001), orgasm (*F* = 31.151; *p* < 0.001), satisfaction (*F* = 23.917; *p* < 0.001), and pain (*F* = 29.025; *p* < 0.001) showed significant differences with time. *Post-hoc* analysis revealed a significant decrease in all subdomains of FSFI between T1 and T2 (all *p* < 0.001), and a significant difference in arousal (*p* < 0.001) and lubrication (*p* = 0.015) was only presented between T1 and T3 (Figure 3).

**Figure 4:**
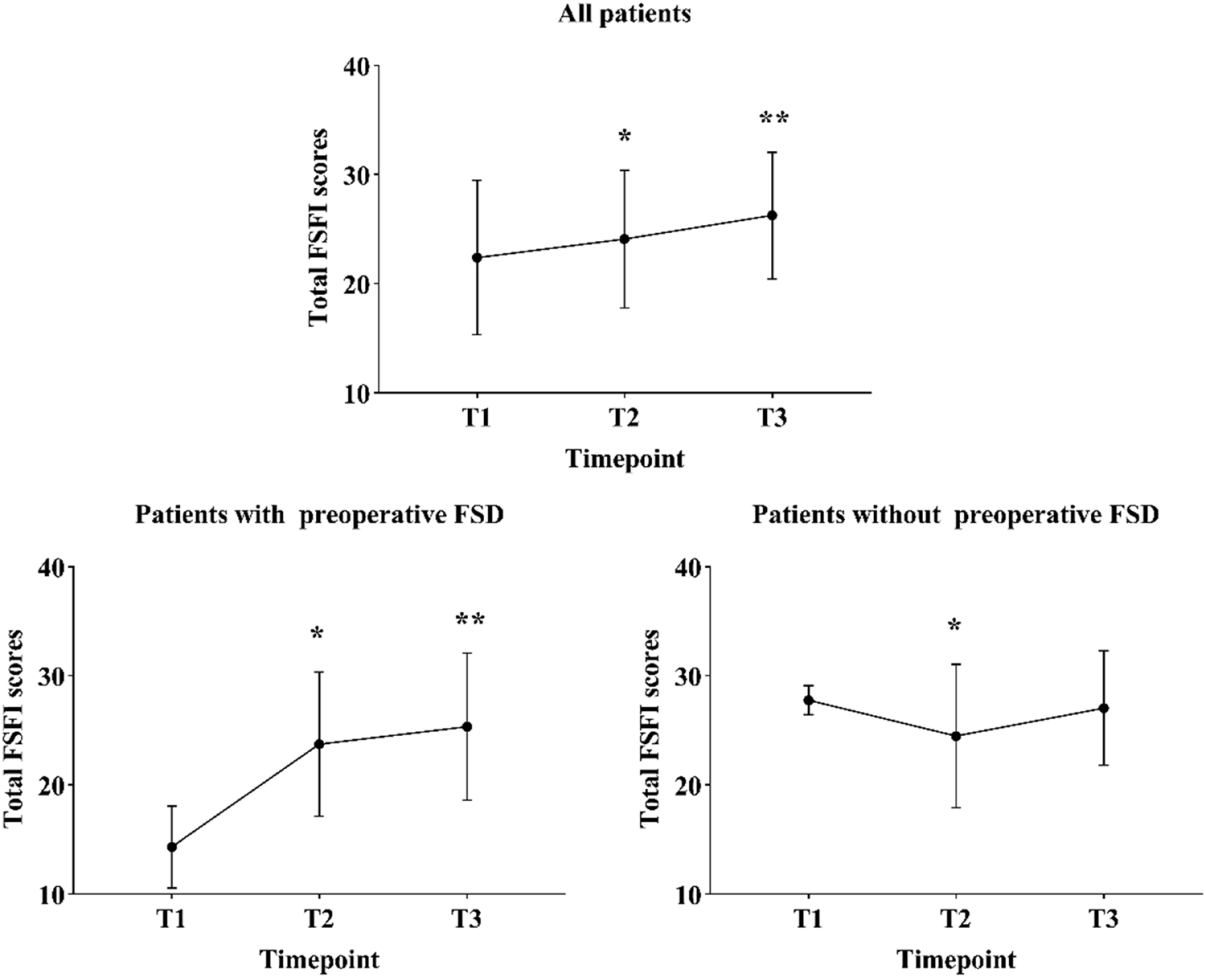
Changes of the total FSFI scores in all patients, patients with pre-FSD and those without pre-FSD. Abbreviations: FSD, female sexual dysfunction; T1, pre-operation; T2, 6 months post-operation; T3, 12 months post-operation. *p < 0.05 represents the difference between T1 and T2. **p < 0.05 represents the difference between T1 and T3.

Furthermore, the total FSFI score of all patients increased from preoperative 26.8 to 27.9 at 6 months (*p* = 0.008) and to 29.8 at 12 months (*p* < 0.001) after surgery. The same trend was observed in patients with pre-FSD (*p* < 0.001). However, in patients without pre-FSD, the total FSFI score decreased from preoperative 27.8 to 27.0 at 6 months (*p* < 0.001), but increased to 28.6 at 12 months after surgery, although the magnitude of this improvement did not reach statistical significance (*p* = 0.54) (Figure 4).

## 4 Discussion

To the best of our knowledge, this was the first study to describe the changes in FSF after LESS hysterectomy. The main findings of the present study are summarized as follows: (1) for all patients, each subdomain and total FSFI scores increased at 6 and 12 months compared with that before operation, (2) for all patients, the prevalence of FSD decreased significantly at 6 and 12 months after surgery, and (3) for patients with pre-FSD, each subdomain and total FSFI scores increased at 6 and 12 months; however, in patients without pre-FSD, FSFI scores decreased at 6 months and no difference was shown at 12 months.

As minimally invasive surgical methods, LESS hysterectomy has demonstrated several advantages in a variety of gynecological conditions, such as reduced postoperative pain, faster recovery, and long-term cosmesis [16,17]. Several studies showed LESS approach could be considered a valid alternative to standard laparoscopy for early-stage endometrial cancer staging due to the comparable peri-operative outcomes [18,19]. However, frailty is prevalent among elderly patients with endometrial cancer [20], which refers to a state of age-related decline in biological reserve, decreased ability to maintain physiological balance, and increased vulnerability to adverse health events. A retrospective cohort study showed that most of the frailty patients had to receive de-escalated treatment [21]. As a consequence, LESS may probably provide an additional treatment decision-making for frailty in patients by decreasing the complication rates.

FSF is a challenging issue that can be affected by a multitude of conditions, some of which are racial, religious, educational, hormonal, physical, psychological, and medical [22]. Historically, for a woman, the uterus has been accepted as a sexual organ, regulating and controlling critical physiological functions [23]. Hence, patients undergoing hysterectomy usually suffer from serious concerns about sexuality, which increases the preoperative stress. Hysterectomy has been under investigation for its effects on female sexuality for quite a long time. However, there is still uncertain about the effects of hysterectomy on FSF.

Up to 37% of the patients with benign disorders exhibited a worsening sexual function following hysterectomy [24]. Goktas et al. [25] observed that total hysterectomy for benign diseases led to a deterioration in sexual function, which was assessed using the FSFI. There are several hypotheses that can explain the deteriorated sexual dysfunction following hysterectomy. The blood supply may decrease after hysterectomy due to loss of female genital organs, consequently resulting in reduced arousal [26]. Another hypothesis is that hysterectomy may cause a reduction in sensibility by interrupting nerve supply, leading to decreased arousal or dyspareunia. Moreover, a decrease in lubrication due to loss of cervix, formation of scar tissue in the upper part of the vagina, and shortening of vaginal length may also affect the FSF [27]. However, there is evidence suggesting that hysterectomy performed for benign diseases may improve FSF [28], which may be explained by relieving symptoms such as dysmenorrhea, dyspareunia and uterine bleeding, and the excision of pelvic lesions alleviating dyspareunia [29].

The data on the influence of hysterectomy types on FSF are contradictory. A study comparing postoperative prevalence of orgasm, frequency, and desire after different types of hysterectomy including vaginal hysterectomy (VH), total laparoscopic hysterectomy (TLH), and total abdominal hysterectomy (TAH) did not display any differences between three groups [22]. Similarly, a study indicated that improvement of sexual function following hysterectomy was irrelevant to surgical techniques used [30]. However, Kiremitli et al. [31] compared the impacts of hysterectomy types in VH, TLH, and TAH approaches on FSF, and observed that sexual function was improved after TLH, which was the best hysterectomy method for preserving sexual function. From this point of view, it is possible to infer that the different surgical procedures may have different effects on surgical outcomes. It has been proved that the vaginal length after hysterectomy is one of the factors affecting sexual satisfaction, and the risk of FSD increases 69.88 folds for each 1 cm shortening in vaginal length [32]. Laparoscopic route has been demonstrated to be more potential than open route in preserving the vaginal length, due to the use of intraoperative uterine manipulator [33]. Our results showed that sexual function in patients who underwent LESS hysterectomy was improved, probably, due to the lower postoperative pain intensity, smaller size of scar, and shorter recovery after surgeries, which was associated with positive effects on body self-perception and a small impact on the patient’s psychogenic sexual function [34]. In addition, the improvement might be explained by the elimination of symptoms such as dysmenorrhea and uterine bleeding [29].

FSD is a multifactorial problem that is defined as a persistent or recurrent disorder of sexual desire, arousal, orgasm, and pain. Possible predictive factors for FSD after hysterectomy are yet to be identified. It was emphasized that age, depression, and relationship problems might affect development of sexual dysfunction after hysterectomy [35]. Dedden et al. found that sexual dysfunction before hysterectomy and marriage or living together were predictive factors for a lower FSFI score [36]. Preoperative FSD was a significant risk factor for postoperative FSD, regardless of the type of hysterectomy with or without oophorectomy [37]. A notable finding of our study was that LESS hysterectomy might significantly improve sexual function in patients with preoperative FSD. We speculated that the status of preoperative sexual function was related to the postoperative changes: the lower the sexual function score before surgery was, the stronger the improvement was [11,38].

This study provided some insights into the changes in sexual function before and after LESS hysterectomy. Although lack of a control group, our study demonstrated that patients undergoing LESS hysterectomy obtained an improvement in sexual function postoperatively; however, it was uncertain to what degree this resulted from simply performing a surgery as opposed to benefits unique to the surgical approach. Besides, psychological factors were not assessed before and after surgery, leading to failure in determining their potential influence on the results. In the future, prospective, multi-center studies with larger sample size are needed to confirm our findings.

## 5 Conclusions

LESS hysterectomy has a significant positive effect on the overall sexual function in women with benign gynecologic diseases, especially those with preoperative sexual dysfunction.
